# Granulosa cell tumor of the ovary and antecedent of adjuvant tamoxifen use for breast cancer

**DOI:** 10.1186/1477-7819-8-67

**Published:** 2010-08-12

**Authors:** Halima Abahssain, Mouna Kairouani, Robert Gherman, Hind M'Rabti, Hassan Errihani

**Affiliations:** 1 Service of medical oncology, National institute of oncology, Rabat, Morocco; 2 Division of Maternal and Foetal Medicine, Prince George's Hospital Centre, Cheverly, USA

## Abstract

**Background:**

Adult granulosa cell tumor associated with antecedent use of tamoxifen as adjuvant hormonotherapy for breast cancer is rare. The pathogenesis of this occurrence remains difficult to explain. The estrogenic effect of tamoxifen can be one such explanation.

**Case presentation:**

A 47 year-old women was treated with surgery, chemotherapy, radiotherapy and tamoxifen for stage III estrogen receptor positive breast carcinoma. Ten months after stopping tamoxifen, we diagnosed a stage Ic granulosa cell tumor of the ovary.

**Conclusions:**

Use of tamoxifen has been found to be associated with gynecological tumors like endometrial carcinoma. Its association with granulosa cell tumor of the ovary is uncommon. Only two previous cases have been reported in literature.

## Background

Granulosa cell tumor is a relatively uncommon ovarian neoplasm accounting for 1% to 2% of all ovarian tumors[1]. Tamoxifen is a non steroidal triphenylethylene that competitively antagonizes the binding of estradiol to estrogen receptor-positive breast carcinoma[2-5]. The occurrence of granulosa cell tumors in patients with antecedent tamoxifen has been previously reported in two patients[6,7]. Although tamoxifen has anti-estrogenic properties, it is converted to several metabolites that can act as estrogen agonists [6]. We report the third case of granulosa cell tumor of the ovary associated with tamoxifen use for breast carcinoma.

## Case presentation

A 47-year-old gravidia 3, para 3 women with chemotherapy induced menopause, had been diagnosed with stage III(T2 N2M0) infiltrating ductal carcinoma of the right breast 5 years before the development of a bilateral granulosa cell tumor of the ovary. After Patey's mastectomy, patient has received 6 cycle of anthracycline based chemotherapy (AC 60 protocol: adriamycin at 60 mg/m^2 ^and cyclophosphamide at 600 mg/m^2^) and adjuvant radiotherapy at a cumulative dose of 50 Gy. The estrogen receptors were strongly positive and the patient received tamoxifen at a dose of 20 mg as single daily dose since January 2004. The tamoxifen was stopped after 5 years. In February 2009, one month after that the patient has stopped the tamoxifen therapy, a nodule in the left ovary was discovered during a laparoscopy for tubal ligation. A left oopherectomy was done, the pathological results showed a granulosa cell tumor. Following this a total hysterectomy, right salpingo-oophorectomy, omentectomy, pelvic and peritoneal washings and multiple peritoneal biopsies were done. The histopathological analysis showed a granulosa cell tumor of the right ovary with capsular rupture in the left ovary, moderate nuclear atypia and mitotic activity ranged between 3 and 5 per ten high-power-fields. Histological evaluation of uterus showed a proliferative endometrial lining. The tumor was classified as stage Ic according to the FIGO classification. The case of our patient was discussed in the multidisciplinary meeting and it was decided to keep her on a close follow-up. Thirteen months after initial diagnosis of granulosa cell of the ovary, she is free of disease.

## Discussion

The granulosa cell tumor is a relatively uncommon ovarian neoplasm accounting 1% to 2% of all ovarian tumors [1]. These lesions occur most frequently in menopausal or postmenopausal women and may be associated with symptoms of estrogen or progesterone secretion [1]. The relationship between this hormonal treatment and the occurrence of such an ovarian tumor is questionable, as so many patients worldwide receive tamoxifen therapy and only two previous cases has been reported in literature till date. It's probable that the association of granulosa cell tumor and the use of tamoxifen for breast cancer is just a random observation and there is no relationship between them.

As mentioned earlier, the granulosa cell tumors in conjunction with tamoxifen administration for breast cancer have been reported in literature. The first case was reported in 1994 by Gherman *et al *[6] in a 52-year-old woman. This patient had liver dysfunction induced by tamoxifen. The authors suggested that the impaired hepatic metabolism of the tamoxifen may be responsible for the ovarian tumor in their patient with elevated liver transaminase levels. The second case was reported in 2002 by Arnould *et al *[7]; they described a case of metastases of a breast carcinoma to an adult granulosa cell tumor in a 63-year-old woman receiving tamoxifen therapy with a past history of breast carcinoma. No explanation was provided for the occurrence and that was only the second case in the literature despite that the tamoxifen being used by a large number of patients around the world with breast cancer. Tamoxifen is an anti-oestrogenic non-steroidal compound widely used for adjuvant therapy in breast cancer [8]. Its proven efficacy as a chemotherapeutic agent has led to its prophylactic use in the prevention of breast cancer in healthy women at high risk of developing breast cancer and it has also shown efficacy in this regard [9]. Despite these anticarcinogenic properties, tamoxifen is also a carcinogen. Women, who take tamoxifen, whether therapeutically or prophylactically, are at significantly increased risk of endometrial cancer [9-11]. Tamoxifen is also a potent liver carcinogen in male and female rats [12], and induces uterine tumors when administered to neonatal [13] and adult rats [14,15]. These findings suggest an appropriate surveillance of these patients treated with tamoxifen in order to proceed to an early diagnosis of secondary gynecological cancers.

Tamoxifen is subject to extensive hepatic metabolism. Not surprisingly, several of the metabolites are predominately estrogenic, rather than antiestrogenic. Differences in tamoxifen metabolism among mice, rats, and humans probably contribute to variation among species-agonist versus partial agonist properties [16].

The metabolite E is generated by the catabolism of tamoxifen, which has a lower affinity for estrogen receptors than tamoxifen [17-19]. However the cis isomer of metabolite E (tamoxifen with a hydroxyl group in place of the dimethylaminoethane side chain) is a potent agonist that displays a high affinity for the estrogen receptor [5,20]. This metabolite has been isolated from dog bile, a species where tamoxifen is predominantly estrogenic. Wiebe *et al *have identified metabolite E and bisphenol in tamoxifen resistant MCF-7 human breast tumors implanted in athymic nude mice, as well as in tumors isolated from patients who have undergone unsuccessful tamoxifen therapy [21,22]. Tamoxifen is known to exhibit estrogenic effects in other animal species. Tucker *et al *were showed in the studies of tamoxifen oncogenicity an elevated of the incidence of granulosa cell tumors at 36% in two groups of female mice receiving 5 or 50 mg/kg of tamoxifen [23]. In their study on cultured rat granulosa cells, welsh *et al *proved that tamoxifen exerts an augmentative, dose-dependent estrogenic effect on FSH-stimulated aromatase activity and estrogen production. They demonstrated that tamoxifen compete with [3H] estradiol for binding to the ovarian estrogen receptors [24].

Raloxifene and tamoxifen are Selective estrogen receptor modulators (SERMs) that have estrogen agonist activities on bone and serum lipid metabolism, and estrogen antagonist activities in mammary tissue in ovariectomized rats [25-27]. Treatment with raloxifene for 6 months resulted in disruption of the hypothalamic-pituitary-ovarian axis, manifested by increased plasma concentrations of luteinizing hormone (LH) and estradiol-17b (E2), and failure of ovulation. Many (56% to 80%) rats in all raloxifene treated groups had focal, minimal to slight hyperplasia of granulosa cells within individual retained follicles. A few treated rats in the mid- and high-dose groups had more extensive focal proliferation of granulosa cells. The results of this study indicate that raloxifene administration to rats causes increases in granulosa cell hyperplasia [25].

## Conclusions

Association between granulosa cell of the ovary and antecedent use of tamoxifen is very rare. When compared to world wide tamoxifen use among women, it appears to be just a random appearance and tamoxifen use does not appear to increase the risk of granulosa cell tumor of the ovary.

## Consent

Written informed consent was obtained from the patient for publication of this case report and accompanying images. A copy of the written consent is available for review by the Editor-in-Chief of this journal.

## Competing interests

The authors declare that they have no competing interests.

## Authors' contributions

HA was responsible for the conception and design for the manuscript, the clinical work, the search for the literature, and the editing work. MK helped in the clinical work as well as the literature review. HM edited the manuscript. HE provided overall supervision and contributed to concept, writing and approval of final version for publication.
